# Autobiography of Charles Caldwell, M. D., with a Preface, Notes, and Appendix

**Published:** 1855-06

**Authors:** 


ART. III.—*Autobiography of Charles Caldwell, M. D., with a Preface, Notes, and Appendix:* By HARRIOT W. WARNER. Philadelphia: Lippincott, Grambo & Co., 1855.

An autobiography offers perhaps the only instance in which the reviewer is privileged to bestow his attention on the personal qualities of the author rather than the intrinsic merits of the book. Published, as this volume is, posthumously, it removes what little force the foolish adage "*de mortuis nil nisi bonum*" might otherwise have, and places, not the auctorial, but the personal and moral attributes of the writer in a position where criticism is fair, where censure, if deserved, cannot be censured again.

It requires no little hardihood for a distinguished man to lay bare the motives and springs of action of a long life, and to leave them as a target for comment after his own departure, and we own that when we heard that Dr. Caldwell had left behind him a book which perpetuated his enmities beyond his death, and deprived him of that shield from attack, the sod that covers his grave, we were astonished that any man could thus give immortality to all those hatreds and jealousies which a long life of ambitious struggle had engendered, and which most men would have gladly buried with them. A careful perusal of the book before us has solved the enigma. Rich in anecdote, stirring in description, and often intensely interesting in its portraitures of the characters of cotemporaries, the work is one to attract the medical mind, and secure a perusal of its entire contents. And it is impossible to withhold from its hero a degree of admiration, excited by his intense individuality, amounting to egotism, his wonderful energy and directness of purpose, and the rude, strong way in which, throughout, he marches on to the accomplishment of his ends.

Born before the American Revolution on the western frontier of North Carolina, Caldwell was even in his boyhood a recluse and a student. In his very childhood he seems to have manifested that quality which in later years we recognize as the governing element in his character. Throughout the work he denies, impliedly, any obligations to the social duties of life. Absorbed in what he deemed his duty to *himself*, he evidences an entire abrogation of his duty to others. Not for him were the amenities of daily intercourse with his fellow man, the pleasures of society, or the softening charms of friendship. Scorning them himself, he scorned all others who differed from him in the estimate of their value.

Passing over the history of his early youth, except to note that the few intimacies he then formed seem to have been interrupted and embittered by an unyielding spirit on his part, we find him in the fall of 1792 a student of medicine in the University of Pennsylvania. He avoided the intercourse of his fellow students, and to this end embarrassed his narrow means that he might occupy a room alone. Punctual at lectures, studious without intermission, sleeping but three or four hours a day, he seems to have resolved on first taking his seat in the lecture room, to some day occupy the chair then filled by Rush. His preceptors at that time were Rush, Shippen, Wistar, Kuhn, Hutchinson, and Griffitts, and of all of these he gives characteristic sketches, and it is singular that in speaking of their personal qualities, he seems most impressed with Kuhn, the cold, icy, precise, unsociable repeater of Cullen's Outlines, who finally left his chair under the odium of wholesale plagiarism.

Medical science at that day was as doctrinal as "foreordination and decree;" *schools* of medicine existed in the form of Brunonianism, Rushism, Tullyism, and for aught we know a dozen other isms. Dr. Caldwell represents Rush, and so far as we can judge, correctly, as being ambitious of occupying the position of head of a band of disciples. Whether he did, as asserted, use his position and personal gifts as inducements to attach students to him, first as a man, and consequently as a medical doctrinarian, we cannot judge from the evidence before us. To this motive Dr. Caldwell attributes certain advances made to him by Rush. This seems at first entirely probable, until a further acquaintance with our author informs us that he himself never made an acquaintance, or advances toward one, without some direct personal gain in view. Of course one acting on this principle assigns to others the same motive. Dr. Rush is described as possessing great social charms. We consider the fact in itself as presumptive evidence of goodness of heart, and honesty of purpose. Exceptions exist, but they *are* exceptions.

All those with whom Caldwell was thrown in contact seem to have been impressed with the acuteness of his intellect, and his indomitable purpose to excel. But one by one we find his friends dropping from him, either in coldness and indifference, or open enmity. His life was one series of antagonisms. In the Philadelphia Medical Society, while yet a student, he triumphed over the older members of the profession in a debate upon the origin of yellow fever. But he was not content to triumph in the argument. A mere boy, he lashed the older men of the profession into a rage which only subsided into permanent enmity, by allusions to their personal conduct during a recent epidemic, which, however true, it was both imprudent and unnecessary to assail. And yet, as an octagenarian, he writes the history of this affair, and repeats the insults then offered, as if to prove that the blood of age was not yet icy, and that he cared less about the success of his doctrines, than of his own exaltation.

Rush had been kind to him. But the time for quarrel soon came. He wrote to Rush describing the effect a wetting in a shower had in allaying an ephemeral fever in his own person. Rush used the fact in his lectures, without credit to Caldwell as the author, and out of this sprang an enmity which resulted in high words at his final examinatian, in Rush's refusal to sign his diploma, and in Caldwell's bold assurance that he could do very well without the name. It was finally arranged, Rush's signature was added to the parchment, but no good feeling existed afterwards. And these were the means which he took to obtain a professorship in his Alma Mater, and failing in that end, claimed to be deprived of his deserts!

In 1819, after more than twenty years residence in Philadelphia, during all which time he was chafing under a sense of neglect, he was invited to the "premiership" of the medical department of Transylvania University. With a dispirited band of colleagues he commenced his labors, and pushing them with an energy yet unbroken, he claims as his own the success of the Lexington school, in which he labored for a period of eighteen years, and then, becoming convinced of the superiority of Louisville as a location, he suddenly abandoned with his colleagues the Lexington enterprise, and was one of the founders of the Louisville Medical Institute, which became subsequently the medical department of the University of Louisville.

And here he taught until the close of the session of 1848—9.

The concluding years of his connection with this school are hurried over in the autobiography, and constitute the most unpleasant portion of the volume; unpleasant to the reviewer, and doubly so to those who are alluded to in its pages, and compelled to defend themselves from its attacks. We shall speak of these occurrences as they appear in Dr. Caldwell's story, and not as they may have been described to us by others. Throwing aside the personal attacks upon his colleagues, and more especially Prof. Yandell, who as his successor was the natural recipient of the largest share of invective—throwing these aside as likely to be the results of prejudice, we find the evidence barren of all but one fact, and that is, that the Trustees requested him to resign and accept an emeritus professorship, assigning his extreme age as their reason for so doing.

Dr. Caldwell graduated in 1794. More than half a century had elapsed since his vigorous quarrel with Prof. Rush. Old age had crept upon him unawares, and the gradual failure which others had noticed was unknown to him. That strong individuality, which we have insisted upon as the leading element of his character, forbade him to compare himself with other men. "I am what I am" was the language of his life.

He looked upon his professional existence as sixty years of continued battle. During all that time he had never yielded a hair to any living man. Those who could not accommodate themselves to him he cast aside; as for him, he was the mountain, and Mahomet must approach. The solemn and instructive fact that in all his professional intercourse he had never formed a friendship which could endure the slightest clashing of interest, had for him no lesson. Looking back on all his former associates, he says "there is none good, no, not one," and wrapped in his unconquerable egotism, he can find nothing in himself to produce these results. That *he* should grow old, that *his* eye should be dimmed, or *his* natural force abated, seemed impossible. And so, when carefully approached and told that the interests of the school required his retirement, all that fire in the old man's nature which age could not quench, was blazing in a splendid paroxysm of anger. No deeper insult could be offered than this. To grow old, superannuated! it was impossible; and he cast the supposition from him as the base invention of rivalry and animosity.

But the thing was inevitable, and the blow fell in its severest form. A dignified retirement, the honors of an emeritus professorship he would not have, and so he finished out the weary remnant of his life in daily denunciation of his old colleagues, and in preparation for its continuance beyond the grave.

He who reads the autobiography of Charles Caldwell will rise from its perusal with a heart-ache. He will recognize the difference between the medical man who makes his profession a source of good to others, who pursues it for the love of science, accepting gratefully what of fame may fall to his lot, who regards himself as one of a great fraternity, with common dignity, and common interests; and he who builds himself up on the downfall of others, who looks upon his profession as merely an avenue to fame, and every way subservient to personal interests.

In this painful autobiography we have, as it seems to us, an instance of the latter, and of its failure to secure its dearest purposes. In the life of Caldwell we see neglect of the social duties resulting in an intense selfishness which poisons every act, and the influences of an unchecked and haughty temper leaving him to die with hardly a friend in that profession in which he had been for sixty years a leader. Genius, energy, application, all were insufficient to counterbalance the faults of the heart.

If we have spoken harshly of one whom we never knew nor saw, and dealt irreverently with an intellect which can never vindicate itself, the circumstances under which this work appears, and the deep moral it inculcates, must be our apology. The magnificent head, the keen and subtle eye, the firm, compressed lips, and the long and flowing beard of snowy whiteness, are hid beneath the coffin lid. Alas! why could he not have buried his enmities in the same grave, and left to men the memory of his brilliant mind, rather than this petty record of his quarrels!

